# Multimodal PET/MRI Imaging Results Enable Monitoring the Side Effects of Radiation Therapy

**DOI:** 10.1155/2018/5906471

**Published:** 2018-11-01

**Authors:** Noémi Kovács, Krisztián Szigeti, Nikolett Hegedűs, Ildikó Horváth, Dániel S Veres, Michael Bachmann, Ralf Bergmann, Domokos Máthé

**Affiliations:** ^1^CROmed Translational Research Centers, H-1047 Budapest, Hungary; ^2^Department of Biophysics and Radiation Biology, Semmelweis University, H-1094 Budapest, Hungary; ^3^Helmholz-Zentrum Dresden-Rossendorf, Institute of Radiopharmaceutical Cancer Research, 01328 Dresden, Germany

## Abstract

Radiotherapy is one of the most frequently applied treatments in oncology. Tissue-absorbed ionizing radiation damages not only targeted cells but the surrounding cells too. The consequent long-term induced oxidative stress, irreversible tissue damage, or second malignancies draw attention to the urgent need of a follow-up medical method by which personalized treatment could be attained and the actually dose-limiting organ could be monitored in the clinical practice. We worked out a special hemisphere irradiation technique for mice which mimics the radiation exposure during radiotherapy. We followed up the changes of possible brain imaging biomarkers of side effects, such as cerebral blood flow, vascular endothelial function, and cellular metabolic processes for 60 days. BALB/c mice were divided into two groups (*n*=6 per group) based on the irradiation doses (5 and 20 Gy). After the irradiation procedure arterial spin labeling (ASL), diffusion-weighted imaging (DWI) in magnetic resonance modality and [^18^F]fluoro-deoxy-D-glucose positron emission tomography (FDG-PET) scans of the brain were obtained at several time points (3, 7, 30, and 60 days after the irradiation). Significant physiological changes were registered in the brain of animals following the irradiation by both applied doses. Elevated standard uptake values were detected all over the brain by FDG-PET studies 2 months after the irradiation. The apparent diffusion coefficients from DWI scans significantly decreased one month after the irradiation procedure, while ASL studies did not show any significant perfusion changes in the brain. Altogether, our sensitive multimodal imaging protocol seems to be an appropriate method for follow-up of the health status after radiation therapy. The presented approach makes possible parallel screening of healthy tissues and the effectiveness of tumor therapy without any additional radiation exposure.

## 1. Introduction

Radiotherapy is one of the most widely spread anticancer treatments in the field of clinical oncology. The paradigm of radiotherapy declares the therapeutic effect is based on indirect and direct DNA damages [1, 2]. Although every cell has well-developed repair mechanisms for DNA impairments, the less-differentiated cancerous cells have diminished ability to repair their broken DNA double-strand based on their uncontrolled and fast reproduction. This unique physiological property serves as the base of radiation therapy where the accumulated absorbed dose determines the severity of the evolving damages (slowed down reproduction, necrosis, or cell death) [1].

Shortly after the exposure, enhanced reactive chemical species concentration can be observed. But, the level of these species continues to arise for several days and months [3]. These oxidative changes affect not only the targeted but also the nontargeted cell population and their progenies as well via intercellular communication pathways [1, 4–9]. They often cause from mild to severe inflammation, irritation, fatigue, xerostomia, oral and gastrointestinal mucositis, radiation dermatitis, and cystitis depending on the irradiated region [10]. The persistence of these stressful effects has significant consequences. Among others, they are responsible for long-term health risks of irradiation such as cardiovascular disease, vascular cell damage, neuropathy, and nerve demyelination as well [1–13]. The second malignancies following radiotherapy are also presumably based on oxidative DNA damages of tumor suppressor genes (p53 and Rb) [14, 15].

One of the most frequently occurring severe late side effects of radiation therapy is myelopathy. Radiation myelopathy (RM) is an irreversible impairment of the brain and spinal cord that has received much attention in the last years [16, 17]. In the case of neuro-oncological irradiation treatments, the healthy brain and spinal cord are critical dose-limiting organs during therapy [1, 17–20].

In clinical oncology, use of medical imaging technologies and the introduction of personalized treatment could refine the whole RT protocol. Thus, it offers a chance to minimize radiation-related side effects. The innovation of medical devices, imaging agents, standardized protocols, and imaging analysis allows to noninvasively capture quantitative and qualitative information about intratumoral heterogeneity. This further helps to personalize radiotherapy for each patient. Thus, the application of imaging radiomics in the wider field of oncology involving toxicity, pathology, imaging, blood biomarkers, demographics, genomics, and proteomics related studies could increase the number of quality years of patients' life. Imaging radiomics for personalized cancer therapy promotes cost effectiveness in the long term too [21]. The quality of life and effectiveness in cost is an important aspect of brain radiation treatments, too.

Due to the fact that widespread use of radiotherapy in the clinical routine currently comes without any standardized and routine monitoring protocol for acute side effects, there is high medical need for an early, sensitive, and harmless in vivo diagnostic imaging method to follow up radiotherapy and continuously monitor the status of patients. This is especially true in the case of patients with primary or secondary brain malignancies. Radiotherapy to the brain can lead to the rise of radiation myelopathy both by focused or whole-brain irradiation techniques. Our approach was to develop and to test multimodal image analysis techniques, which are able to investigate the emerging metabolic, perfusion, and diffusion-related changes in the body after the irradiation of neural tissues. To this end, we chose widely accessible imaging methods in magnetic resonance imaging (MRI) and in [^18^F] fluoro-deoxy-D-glucose (FDG) positron emission tomography (PET). FDG-PET and MRI are clinically easily accessible modalities that are usually applied in the work-up of almost all cancer patients especially in cancer patients with brain involvement. We applied a mouse model of partial brain irradiation. Our purposes included detection of neural correlates. Radiation-induced effects reach not only the immediate environment of the tumor but seemingly distant parts and regions of the brain too. To investigate this phenomenon, we applied the image analysis technique of correlations between brain regions. This way, effects of irradiation to, e.g., one hemisphere could be detected in the other seemingly unaffected hemisphere too. We applied two single absorbed dose levels of 5 Gy and 20 Gy to investigate the standalone effect of single doses applied in the clinical practice of multidose fractionated radiotherapy. These two radiation doses were selected to account for the low-dose clinical fraction value and the high-dose clinical fraction value.

## 2. Methods

### 2.1. Animals

All applicable international, national, and institutional guidelines for the care and use of animals were followed, and in particular, all animal experiments were carried out according to the guidelines of German Regulations for Animal Welfare and have been approved by the Landesdirektion Dresden. The experimental procedure conforms in particular to the European Convention for the Protection of Vertebrate Animals Used for Experimental and Other Scientific Purposes (ETS No. 123). 12 BALB/c female mice were divided into two groups (*n*=6) differing in irradiation doses (5 and 20 Gy). Animals were fed ad libitum and maintained under controlled temperature, humidity, and light conditions.

### 2.2. Irradiation

**Figure 1:**
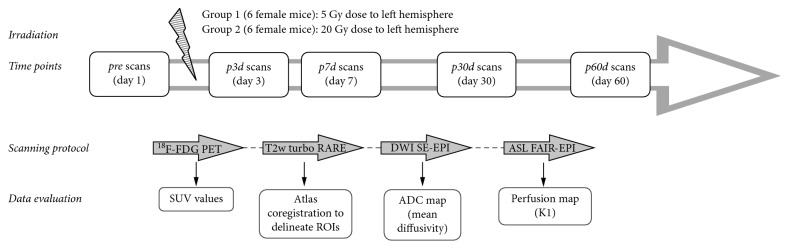
The schedule of irradiation and image data processing.

The irradiation was made under ketamine/medetomidine anesthesia. The schedule of our study regarding irradiation and imaging protocols is illustrated on Figure 1.

Instead of whole-brain irradiation, the left hemisphere irradiation was chosen. For reproducible positioning, a custom-made mouse holder and lead collimator were used. The holder consisted of a plastic box with lead covering on the top and ear and teeth sticks for fixing the position of the mice. The irradiated area (0.7 × 1 cm hole in the lead shielding) was defined based on an MRI scan. To avoid the high eye-dose, the eyes were shielded as well. Irradiation was done by an Yxlon X-ray tube (MGC-41 Maxishot, calibrated at 200 kV and 20 mA) with an ambient filter (combination of 3 mm beryllium, 3 mm aluminium, and 0.5 mm copper). Two different irradiation schemes were tested in our experiments, which differed only in the duration of the irradiation, thus in dose of 5 Gy and 20 Gy.

### 2.3. Imaging

Imaging was made under desflurane anesthesia at 5 different time points—before irradiation (“*pre*”), 3 days (“*p3d*”), 7 days (“*p7d*”), 30 days (“*p30d*”), and 60 days (“*p60d*”) after irradiation.

FDG-PET imaging was performed on a nanoScan PET-CT small-animal imaging system (Mediso Ltd., Budapest, Hungary) with a special custom-made animal bed capable of scanning two mice simultaneously. The mice were fastened for 14 hours prior to scanning. 60 minutes before the brain scans, the animals were generally anesthetised with desflurane (9% desflurane in 30% oxygen/air), and 30 min before the scan, 5.05 ± 3.04 MBq [^18^F]FDG was administered intravenously in the tail vein. The imaging parameters were normal mode with packet timestamping and 50% axial overlap. The reconstruction used the Monte Carlo-based OSEM technique with 4 iterations, 3 subsets, a 400–600 keV energy window, 1 : 5 coincidence window, and attenuation correction.

MRI scans were performed on a 7 T small animal MRI system (BioSpec 70/30, Bruker, Germany) equipped with active shielded 200 mT/m gradients and a head surface coil only for receiving. The scanning protocol started with a fat-suppressed and respiratory-triggered T2-weighted turbo rapid acquisition with refocused echoes (TurboRARE) sequence as an anatomical background at the same position as the following scans. The imaging parameters were 16 axial slices, slice thickness of 0.8 mm, gap of 0.2 mm, in-plane resolution of 0.15 mm, TR/TE 4315/45 ms, 4 averages, RARE factor of 8.

Diffusion-weighted imaging (DWI) based on an echo-planar imaging (EPI-SE) sequence had the same geometrical parameters as the anatomical scan except in this case the in-plane resolution was 0.234 mm and TR/TE 3000/31 ms. Diffusion-weighting parameters were chosen to be Δ/*δ* 14/7 ms and *b*-values (0, 100, 200, 400, 600, 800, and 1000 s/mm^2^) in three orthogonal directions.

For the perfusion measurements, the arterial spin labeling (ASL) technique using the flow alternating inversion recovery (FAIR) method was performed with adiabatic hyperbolic secant inversion pulse and echo-planar imaging acquisition (EPI-SE, TR/TE 9000/14.67 ms, 5 averages). This single-shot, multiphase ASL measurement was acquired on a single slice (slice thickness of 0.8 mm, in-plane resolution of 0.18 × 0.225 mm) containing both thalamus and hippocampus, repeated 22 times (minimal inversion recovery time (TIR), 26 ms and 200 ms increment between the successive inversion times).

### 2.4. Postprocessing

**Figure 2:**
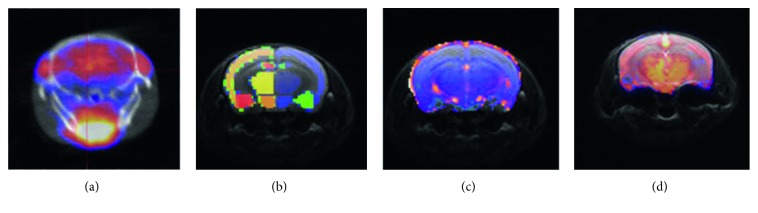
[^18^F]FDG-PET-CT coregistered to MRI images (a). Altas coregistered T2w turboRARE (b). ADC map coregistered to T2w turboRARE (c). Perfusion map coregistered to T2w turboRARE (d) (all images are from individual studies).

The first step of our postprocessing was the manually atlas-based (mouse brain atlas from Brookhaven National Laboratory [22]) registration of each scan using VivoQuant software (inviCRO, USA). The further evaluation of MRI scans to create parametric maps was performed by a self-written code in MATLAB (The Mathworks Inc., USA). Thus, parametric maps could be investigated in the hypothalamus, thalamus, hippocampus, neocortex, amygdala, and striatum separately (Figure 2). Cerebellum was excluded from the evaluation based on the imperfect shimming and EPI distortion on the MRI scans.

In case of [^18^F]FDG-PET scans, activity concentration (*A*_conc_) was calculated in ROIs and the standard uptake value (SUV in g/mm^3^) as a measure of [^18^F]FDG uptake was determined as follows:

(1) $$\begin{array}{c} \text{SUV}=\frac{A_{\text{conc}}}{\left(A_{\text{inj}}/\text{BW}\right)}, \end{array}$$

where *A*_inj_ is the decay-corrected injected activity and BW is the body weight of the mouse. Based on the diffusion-weighted scans, the apparent diffusion coefficient (ADC) map was calculated with a voxel-wise monoexponential curve fitting in all three diffusion weighting directions. Not to incorporate direction-dependence, the mean ADC value was determined as the mean of the three apparent diffusion coefficients corresponding to the different orthogonal diffusion-weighting directions.

In the ASL, a scan evaluation one-tissue compartment model was used to describe the transport kinetics of water molecules accounting for the limited membrane permeability of the blood-brain barrier (BBB) [23]. The calculated *K*_1_ map (equivalent with the uptake rate) practically equals to the blood flow if the BBB is considered perfectly permeable for water:

(2) $$\begin{array}{c} K_{1}=V_{\text{d}}\;*\;R_{1,a}\frac{R_{1,s}-R_{1,\text{ns}}}{R_{1,\text{ns}}}, \end{array}$$

where volume of distribution of water in tissue (*V*_d_) is considered to be known in our model *V*_d_ = 0.95 mLH_2_O/mL [24]. *R*_1,*i*_ is the reciprocal of longitudinal relaxation rates, respectively, to the arterial water (*a*), selective scan (*s*), and nonselective scan (ns). The last two were determined voxel-wise by least square fitting of

(3) $$\begin{array}{c} S_{s,\text{ns}}=c+\left|S_{0}\left(\frac{1-2eT_{1}}{R_{1s,\text{ns}}}\right)\right|, \end{array}$$

where *S* is the MRI signal of the selective or nonselective FAIR scan, *c* is a bias, *S*_*0*_ corresponds to the proton density, and *T*_1_, *a* is the relaxation rate of arterial water—in our model, *T*_1_, *a*=2*s* [25].

### 2.5. Data Analysis

For statistical analysis, the mean MRI signals and calculated SUV, ADC, and *K*_1_ values were exported from every ROI. The analysis was performed for all 3 modalities. At first, difference between the left and right whole (the mean of different regions' ROI values was calculated) hemispheres was determined and related to the left hemisphere for each rat at each time point. Thereafter, correlation (using Pearson correlation coefficients) between hemispheres among brain regions was determined regardless the time points. The time trend was analyzed on the whole brain using a linear mixed effect model (random slope and intercept model with quadratic time effect using restricted maximum likelihood calculation) and with controlling for dose (Stata/IC 15.0, StataCorp LLC, Texas, USA).

## 3. Results

### 3.1. FDG-PET

**Figure 3:**
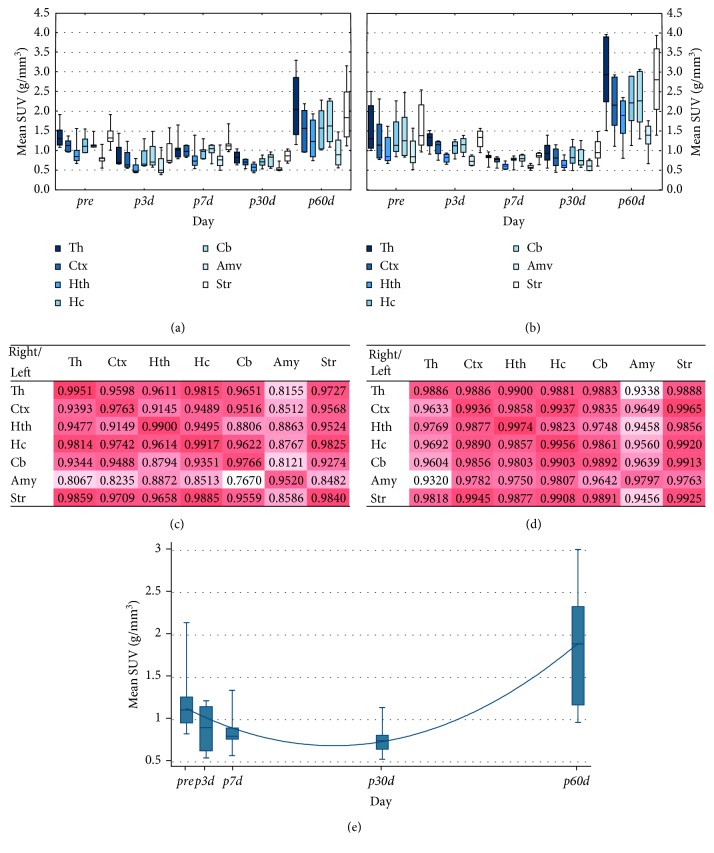
The effect of irradiation on brain [^18^F]FDG uptake values. The mean SUVs of the left (irradiated) brain regions (Th: thalamus; Ctx: cortex; Hth: hypothalamus; Hc: hippocampus; Cb: cerebellum; Amy: amygdala; Str: striatum) are shown at five different time points by 5 Gy (a) and 20 Gy (b) dose groups.The boxplots show median with quartiles, minimum, and maximum. Correlation maps of [^18^F]FDG uptake values of the left versus right brain areas (regardless of the time points) by 5 Gy (c) and 20 Gy (d) dose groups are shown. The heat maps visualize the Pearson correlation of irradiation effects between each investigated brain region. The time trend analysis of the whole brain is shown in (e).

FDG-PET studies were able to register the metabolic changes within the brain of animals.  Figure 3 illustrates the mean SUV values of brain regions at five different time points using 5 Gy (A) and 20 Gy (B). Strong correlation (except the cerebellum and amygdala) was found between contralateral brain regions in either dose groups as shown in Figures 3(c) and 3(d). The relative SUV difference between the hemispheres was only 0.1%; thus the sum of the SUVs was acceptable. The time trend analysis of the whole brain did not show any statistical evidence for difference in the effect of 5 Gy and 20 Gy (*p* < 0.05) but revealed significant (*p* < 0.05) quadratic effect (Figure 3(e)).

Shortly after the irradiation, no relevant [^18^F]FDG uptake changes (in term of mean SUV) could be seen in the investigated brain areas, but two months later, relevant increased SUVs were registered in the whole brain.

### 3.2. DWI

**Figure 4:**
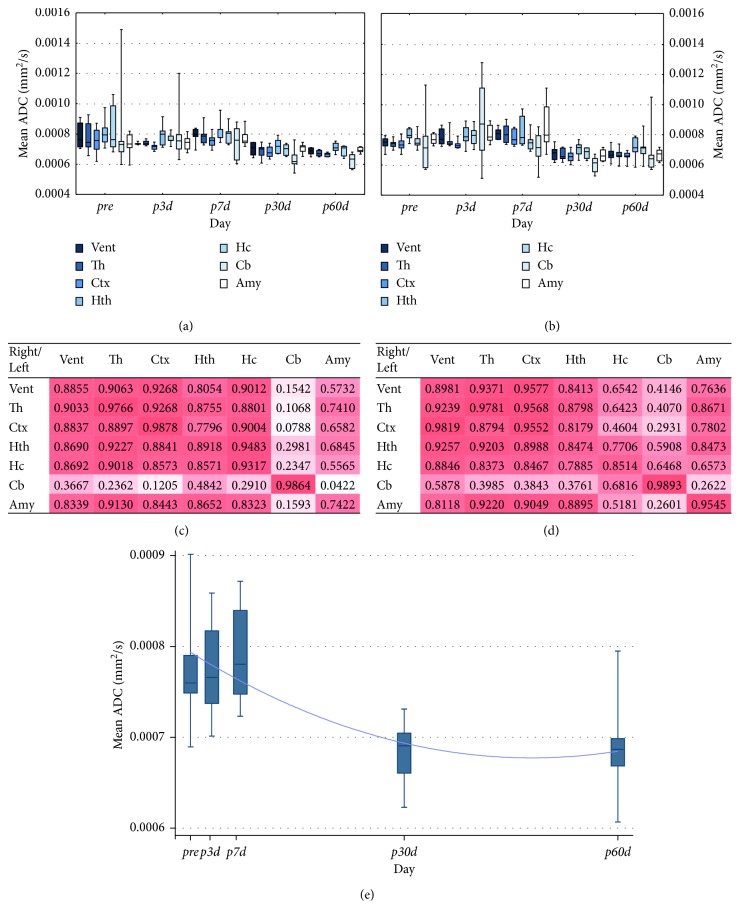
The effect of irradiation on the brain ADC values. The mean ADC values of the left (irradiated) brain regions (Vent: ventricles; Th: thalamus; Ctx: cortex; Hth: hypothalamus; Hc: hippocampus; Cb: cerebellum; Amy: amygdala) are shown at five different time points by 5 Gy (a) and 20 Gy (b) dose groups. The boxplots show median with quartiles, minimum, and maximum. The correlation maps of the ADC values of the left versus right brain areas (regardless of the time points) by 5 Gy (c) and 20 Gy (d) dose groups are shown. The heat maps visualize the Pearson correlation of irradiation effects between each investigated brain region. The time trend analysis of the whole brain ADC changes is shown in (e).

Figure 4 illustrates the mean ADC values of brain regions at five different time points using 5 Gy (A) and 20 Gy (B). Strong correlation was found between contralateral brain regions in either dose groups as shown in Figures 4(c) and 4(d). The relative ADC value difference between the hemispheres was only 1.2%; thus ADC values were summed. The time trend analysis of the whole brain did not show any statistical evidence for difference in the effect of 5 Gy and 20 Gy (*p* < 0.05) but revealed a significant (*p* < 0.05) quadratic effect (Figure 4(e)).

As the fitted trend line shows no ADC value, changes were detected at the first week. One month after the irradiation, relevant ADC value decreasing was registered in all brain regions, whose values did not alter until the end of the measurement (Figure 4(e)).

### 3.3. ASL

**Figure 5:**
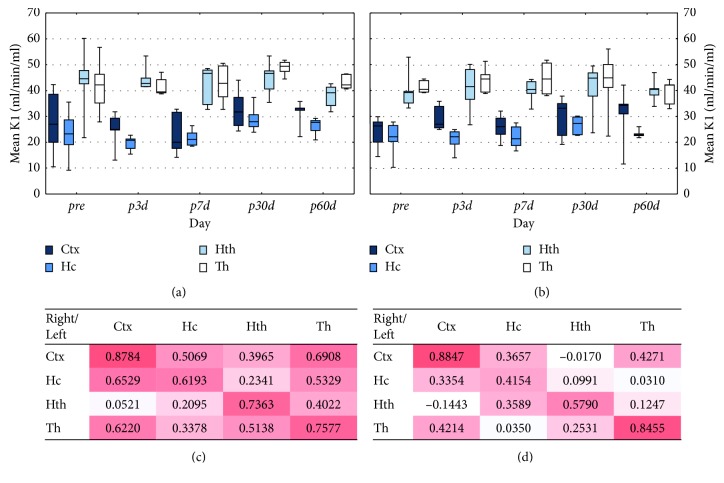
The effect of irradiation on the brain *K*_1_ values. The mean *K*_1_ values of the left (irradiated) brain regions (Ctx: cortex; Hc: hippocampus; Hth: hypothalamus; Th: thalamus) are shown at five different time points by 5 Gy (a) and 20 Gy (b) dose groups. The boxplots show median with quartiles, minimum, and maximum. The correlation maps of the *K*_1_ values of the left versus right brain areas (regardless of the time points) by 5 Gy are presented in (c) and for 20 Gy dose groups in (d). The heat maps visualize the Pearson correlation of irradiation effects between each investigated brain region.

Figure 5 illustrates the mean *K*_1_ values of brain regions at five different time points using 5 Gy (A) and 20 Gy (B). Moderate correlation was found between contralateral brain regions in either dose groups as shown in Figures 5(c) and 5(d). The relative *K*_1_ value difference between the hemispheres was 0.8%; thus the ASL values were summed. The time trend analysis of the whole brain did not show any statistical evidence for difference in the effect of 5 Gy and 20 Gy (*p* > 0.1). There was no significant trend in time (*p* > 0.1).

## 4. Discussion

We have investigated the short- and long-term risks and side effects of radiation therapy to the brain. In this study, a custom-made small animal irradiation system using two different doses mimicked the effects of high- and low-dose single RT fractions in clinical practice. The applied multimodal imaging method has monitored important physiological parameters (cerebral blood flow, vascular endothelial function, and cellular metabolic processes) for 60 days.

The 5 Gy and 20 Gy single-fraction radiation doses are equivalents of the single dose of the lowest and the highest human doses during a fractioned RT [26].

[^18^F]FDG-PET scans made possible to follow up brain glucose use of animals after the irradiation [27]. The [^18^F]FDG uptake and the calculated SUV strictly correlate with local glucose metabolism, reflecting different constituents of brain glucose uptake: glial metabolism, neuronal and synaptic activity, and local immune processes [28]. As such, [^18^F]FDG could be used to monitor eventual microglial or other inflammatory conditions in the brain postradiation therapy too.

Diffusion-weighted MRI has been used for the monitoring of physiological changes (oedema, inflammation, fibrosis, and necrosis) within the brain tissue and for the quantification of MRI signal loss using ADC. ADC as a clinically validated measurand in the field of oncology is able to differentiate between the RT-induced tissue changes. Malignant tumor disease is often described with lower ADC values while oedema and inflammation are manifested in higher ADC values [29, 30]. Besides these changes, decreased ADC values were reported after RT in case of noncancerous tissues by Takayama et al. [31].

In our study, a trend of decrease in ADC of both the irradiated and the correlating hemispheral brain structures could be observed starting a month after radiation therapy and it was maintained up to the end of the 60 day observation period. Our results of ADC values are close to these and other previous findings [32–34]. The effect of RT is assumed to be related to the damage of vascular endothelial cells, which influences the swelling of the concerned cells and decreases the extracellular space in case of inflammation.

The arterial spin labeling technique, as a proposed alternative of PET scan, has the potential for detection and quantitative follow-up the cerebral blood flow changes without any contrast material [24]. Following RT, there is a “latent period” when no significant perfusion and vascular changes could be registered. However, the length of this period is absolutely depending on the applied doses which vary from a few months to a few years. 20 Gy dose whose effect on rat brain equivalent with the tissue response to a mean total dose of human-fractionated brain RT—applied by Reinhold et al. caused significant transient neurotransmitter enhancement, increased CBF, and decreased extravascular space in rats 3 months after the RT [35]. In our 2 month long experiments, the animals did not show any perfusion changes in either dose groups (5/20 Gy dose groups). Based on the high intersubject variability, the inherently low signal-to-noise ratio (SNR) of this technique and the potential presence of “latency period” after irradiation showing up in perfusion changes more animals, and a longer follow-up period would have been necessary to observe eventual trends in perfusion changes in the brain with MRI.

No hemisphere-related changes were observed in the case of 5/20 Gy dose groups using either FDG-PET and DWI MRI. However, statistical trend analysis could detect the methods' applicability for monitoring course of the side effects. Interestingly, the results of correlation analysis of regions show a high correlation between the hemispheres. This may indicate long-distance bystander side effects of radiotherapy in the CNS. The detectability and effects of irradiation between the 5–20 Gy dose range were not different which draws the attention to an existing side effect profile of even low-dose fractionated radiotherapy.

## 5. Conclusions

We succesfully developed a new multimodal imaging protocol which is able to monitor the irradiation-related side effects and follow up the health status of the animals for several weeks. The most relevant imaging biomarkers in the field of neuroscience were able to responsibly indicate the forming side effects of RT. Elevated brain glucose consumption and decreased diffusion with nearly constant cerebral blood flow were the first detectable signs of the altered local circulation and transportation, the changed metabolic activity, and the induced inflammation by reactive oxygen species (ROS) production.

Based on the high translational power of this quantitative MRI/PET technique and the availability of these modalities in clinics, it is assumed that our scientific results could support and refine the radiation therapy via the monitoring of side effects and follow up the health status of patients. In addition, this imaging protocol can support the realization of personalized therapy in medical practice.

Hereby, a new multimodal imaging protocol is presented which the authors consider an appropriate monitoring method to follow both effects of irradiation of the tumor and side effects of radiation therapy.

## Data Availability

The data used to support the findings of this study are available from the corresponding author upon request.

## References

[1] Azzam E. I., de Toledo S. M., Little J. B. (2003). Oxidative metabolism, gap junctions and the ionizing radiation-induced bystander effect. *Oncogene*.

[2] Hall E. J., Giaccia A. (2006). *Radiobiology for the Radiologist*.

[3] Petkau A. (1987). Role of superoxide dismutase in modification of radiation injury. *British Journal of Cancer. Supplement*.

[4] Spitz D. R., Azzam E. I., Li J. J., Gius D. (2004). Metabolic oxidation/reduction reactions and cellular responses to ionizing radiation: a unifying concept in stress response biology. *Cancer and Metastasis Reviews*.

[5] Kryston T. B., Georgiev A. B., Pissis P., Georgakilas A. G. (2011). Role of oxidative stress and DNA damage in human carcinogenesis. *Mutation Research/Fundamental and Molecular Mechanisms of Mutagenesis*.

[6] Tamminga J., Kovalchuk O. (2011). Role of DNA damage and epigenetic DNA methylation changes in radiation-induced genomic instability and bystander effects in germline in vivo. *Current Molecular Pharmacology*.

[7] Hei T. K., Zhou H., Chai Y., Ponnaiya B., Ivanov V. N. (2011). Radiation induced non-targeted response: mechanism and potential clinical implications. *Current Molecular Pharmacology*.

[8] Seymour C. B., Mothersill C. (2004). Radiation-induced bystander effects-implications for cancer. *Nature Reviews Cancer*.

[9] Prise K. M., O’Sullivan J. M. (2009). Radiation-induced bystander signalling in cancer therapy. *Nature Reviews Cancer*.

[10] Hall E. J., Giaccia A. J. (2018). *Radiobiology for the Radiologist*.

[11] Cucinotta F. A., Chappell L. J. (2010). Non-targeted effects and the dose response for heavy ion tumor induction. *Mutation Research/Fundamental and Molecular Mechanisms of Mutagenesis*.

[12] Tubiana M. (2009). Can we reduce the incidence of second primary malignancies occurring after radiotherapy? a critical review. *Radiotherapy and Oncology*.

[13] National Research Council (2006). Committee to assess health risks from exposure to low levels of ionizing radiation. *Health Risks from Exposure to Low Levels of Ionizing Radiation: Beir VII Phase II*.

[14] Hendry J. H. (2001). Genomic instability: potential contributions to tumour and normal tissue response, and second tumours, after radiotherapy. *Radiotherapy and Oncology*.

[15] Robles A. I., Linke S. P., Harris C. C. (2002). The p53 network in lung carcinogenesis. *Oncogene*.

[16] Chao M. W., Wirth A., Ryan G., MacManus M., Liew K. (1998). Radiation myelopathy following transplantation and radiotherapy for non–Hodgkin’s lymphoma. *International Journal of Radiation Oncology∗Biology∗Physics*.

[17] Maranzano E., Bellavita R., Floridi P. (2001). Radiation-induced myelopathy in long-term surviving metastatic spinal cord compression patients after hypofractionated radiotherapy: a clinical and magnetic resonance imaging analysis. *Radiotherapy and Oncology*.

[18] Okada S., Okeda R. (2001). Pathology of radiation myelopathy. *Neuropathology*.

[19] Sarica F., Ozgur K., Cekinmez M., Nur A., Kadir T. (2012). Delayed radiation myelopathy: differential diagnosis with positron emission tomography/computed tomography examination. *Asian Journal of Neurosurgery*.

[20] Kempf S. J., Azimzadeh O., Atkinson M. J., Tapio S. (2013). Long-term effects of ionising radiation on the brain: cause for concern?. *Radiation and Environmental Biophysics*.

[21] Lambin P., Rios-Velazquez E., Leijenaar R. (2012). Radiomics: extracting more information from medical images using advanced feature analysis. *European Journal of Cancer*.

[22] Brookhaven National Laboratory; BNL, http://brainatlas.mbi.ufl.edu/

[23] Boś A., Bergmann R., Strobel K., Hofheinz F., Steinbach J., van den Hoff J. (2012). Cerebral blood flow quantification in the rat: a direct comparison of arterial spin labeling MRI with radioactive microsphere PET. *EJNMMI Research*.

[24] Leithner C., Müller S., Füchtemeier M., Lindauer U., Dirnagl U., Royl G. (2010). Determination of the brain–blood partition coefficient for water in mice using MRI. *Journal of Cerebral Blood Flow and Metabolism*.

[25] Dobre M. C., Uğurbil K., Marjanska M. (2007). Determination of blood longitudinal relaxation time (T1) at high magnetic field strengths. *Magnetic Resonance Imaging*.

[26] Pollack A., Ahmed M. (2011). *Hypofractionation: Scientific Concepts and Clinical Experiences*.

[27] Peterson T. E., Manning H. C. (2009). Molecular imaging: 18F-FDG PET and a whole lot more. *Journal of Nuclear Medicine Technology*.

[28] Berti V., Mosconi L., Pupi A. (2014). Brain: normal variations and benign findings in fluorodeoxyglucose-PET/computed tomography imaging. *PET clinics*.

[29] Chenevert T. L., Meyer C. R., Moffat B. A. (2002). Diffusion MRI: a new strategy for assessment of cancer therapeutic efficacy. *Molecular Imaging*.

[30] Ross B. D., Moffat B. A., Lawrence T. S. (2003). Evaluation of cancer therapy using diffusion magnetic resonance imaging^1^. *Molecular Cancer Therapeutics*.

[31] Takayama Y., Kishimoto R., Hanaoka S. (2008). ADC value and diffusion tensor imaging of prostate cancer: changes in carbon‐ion radiotherapy. *Journal of Magnetic Resonance Imaging*.

[32] Kozlowski P., Chang S. D., Jones E. D., Berean K. W., Chen H., Goldenberg S. L. (2006). Combined diffusion-weighted and dynamic contrast-enhanced MRI for prostate cancer diagnosis—correlation with biopsy and histopathology. *Journal of Magnetic Resonance Imaging*.

[33] Kumar V., Jagannathan N. R., Kumar R. (2006). Correlation between metabolite ratios and ADC values of prostate in men with increased PSA level. *Magnetic Resonance Imaging*.

[34] Tanimoto A., Nakashima J., Kohno H., Shinmoto H., Kuribayashi S. (2007). Prostate cancer screening: the clinical value of diffusion-weighted imaging and dynamic MR imaging in combination with T2-weighted imaging. *Journal of Magnetic Resonance Imaging*.

[35] Reinhold H. S., Hopewell J. W. (1980). Late changes in the architecture of blood vessels of the rat brain after irradiation. *British Journal of Radiology*.

